# Thermal and Mechanical Properties of Silica–Lignin/Polylactide Composites Subjected to Biodegradation

**DOI:** 10.3390/ma11112257

**Published:** 2018-11-13

**Authors:** Aleksandra Grząbka-Zasadzińska, Łukasz Klapiszewski, Sławomir Borysiak, Teofil Jesionowski

**Affiliations:** Institute of Chemical Technology and Engineering, Faculty of Chemical Technology, Poznan University of Technology, Berdychowo 4, PL-60965 Poznan, Poland; aleksandra.grzabka-zasadzinska@put.poznan.pl (A.G.-Z.); lukasz.klapiszewski@put.poznan.pl (Ł.K.)

**Keywords:** silica–lignin hybrid materials, polylactide, physicochemical and morphological properties, mechanical properties, biodegradation

## Abstract

In this paper, silica–lignin hybrid materials were used as fillers for a polylactide (PLA) matrix. In order to simulate biodegradation, PLA/hybrid filler composite films were kept in soil of neutral pH for six months. Differential scanning calorimetry (DSC) allowed analysis of nonisothermal crystallization behavior of composites, thermal analysis provided information about their thermal stability, and scanning electron microscopy (SEM) was applied to define morphology of films. The influence of biodegradation was also investigated in terms of changes in mechanical properties and color of samples. It was found that application of silica–lignin hybrids as fillers for PLA matrix may be interesting not only in terms of increasing thermal stability, but also controlled biodegradation. To the best knowledge of the authors, this is the first publication regarding biodegradation of PLA composites loaded with silica–lignin hybrid fillers.

## 1. Introduction

During the last few years, due to the growing environmental concerns regarding waste disposal and increasing prices of fossil fuels, demand for green products obtained from renewable resources has significantly increased. Great deal of interest was generated especially by biodegradable polymers. Among many biodegradable polymers, poly(lactic acid) (PLA) appears to be one of the most attractive materials. PLA is so popular because of renewability of it resources (it can be produced from 100% renewable resources like corn, sugar beets or rice), good mechanical properties, and biodegradability [1]. Apart from these indisputable advantages PLA has also some serious drawbacks that limit its wider application—poor thermal stability, low crystallization ability, and low barrier properties [2]. Nevertheless, it has been found that the above problems can be overcome by addition of fillers.

Silica–lignin hybrid filler combines highly available, low cost kraft lignin and silica with good mechanical and thermal properties [3,4]. The kraft lignin has in its structure numerous hydroxyl groups which are often found problematic and has to be masked or modified [5,6]. These hydroxyl groups are also responsible for biodegradation of lignin [7] and its degradation in temperature over 170 °C [8]. Hence, combining of lignin with a thermostable, inorganic filler such as silica is thought to provide an increased thermal stability. Although intensive research on silica–lignin hybrid fillers is being carried out [9,10,11], there are still not many publications dealing with polymer/hybrid fillers composites [3,12,13,14]. Silica–lignin hybrid coupled with ammonium polyphosphate was tested as a novel intumescent flame-retardant system to improve the fire retardancy of PLA [13]. Bula et al. [3] confirmed that silica–lignin hybrid filler enhances thermal stability of polypropylene (PP) composites. It was also stated that as the percentage content of the hybrid filler in the PP matrix increases, thermal stability of composite increases, too. What is more, based on DMTA analysis, Strzemiecka et al. [12] concluded that the thermo-mechanical properties of the composite containing silica–lignin filler depended on the lignin-to-silica ratio in the hybrid filler. Also, composites consisting of phenolic resin and silica–lignin hybrid filler were characterized with better thermo-mechanical properties than systems with lignin or silica alone [14]. Therefore, application of such hybrid fillers in polymer composites is well-grounded and should be further investigated.

Polymers that biodegrade are already very desirable in agriculture or packaging because they eliminate the waste disposal issue. In case of PLA biodegradation, the most important process is hydrolysis which is catalyzed by end groups of carboxylic acids (autocatalytic reaction). The speed of this process is mainly determined by the molecular weight and stereochemical composition of sample [15,16]. However, it turns out that in case of PLA composites fillers may also have an effect on the biodegradation process of PLA. There are information that addition of starch blends or wood flour to PLA accelerates the thermal decomposition of composites [17]. Blending organically modified montmorillonites with PLA was found to increase the degradation rate [18], while PLA/PEG/nano-silica composites showed similar degradation behavior as PLA/PEG films [19]. Although detailed literature research, no study on biodegradation of PLA/hybrid fillers was found.

In view of the above considerations, it would appear that one of the directions that ought to be taken in order to further develop PLA-based composites, e.g., for packaging applications, is to define how biodegradation process of such materials affects their properties.

For that reason, the aim of this work was to determine changes taking place during biodegradation of PLA composites filled with silica–lignin fillers of different composition. For this purpose, thermal stability, crystallization behavior, change of color, morphology, and mechanical properties of composites before and after simulated biodegradation process were defined. This work is a continuation of our study on PLA composites with silica–lignin hybrid fillers [20].

## 2. Materials and Methods

### 2.1. Materials

Silica–lignin hybrid filler was produced using commercial Syloid 244 silica (W.R. Grace & Co., Columbia, MD, USA) and kraft lignin (Sigma-Aldrich, Steinheim am Albuch, Germany). Polylactide, type Ingeo 2500 HP was purchased at Nature Works, Minnetonka, MN, USA.

### 2.2. Preparation of Silica–Lignin Hybrid Materials

Four silica–lignin hybrid materials containing silica and lignin in weight ratios 1:1, 2:1, 5:1, and 20:1 were prepared in the same way as in previous publications [3,20,21,22]. Later in this paper, silica–lignin hybrid materials will be described as “hybrid”, e.g., 20:1 hybrid stands for silica–lignin in ratio 20:1.

### 2.3. Preparation of Polylactide/Silica–Lignin Hybrid Composites

Composite pellets of polylactide and 7.5% (*w*/*w*) of each hybrid filler were prepared in a co-rotating twin screw extruder (ø = 16 mm, L/D = 40, EHP 1614, Zamak Mercator Sp. z o.o., Skawina, Poland) and then cut with knife mill (25-16/TC-SL, TRIA, Novi, MI, USA). The process parameters for this first extrusion were following: a barrel temperature of 180–200 °C and a screw rotation speed of 145 rpm. Films for further characterization were produced with single screw extruder (ø = 25 mm, L/D = 30, Metalchem, Warszawa, Poland) with slit die and chill-roll puller (pull speed 2.5 m/s). In this second extrusion the process parameters were subsequently 17–220 °C and 80 rpm.

The samples were named according to the following convention: “d” stands for sample after biodegradation, PLA stands for polylactide, and numbers stand for filler type. For example, name “dPLA/20:1”means biodegraded sample of polylactide filled with 20:1 hybrid filler.

### 2.4. Biodegradation of Composite Films

Films were cut into pieces of 10 mm width and 100 mm length and subsequently put into stainless steel mesh envelops. They were buried in the conditioned soil of neutral pH, at 8 cm depth. The initial temperature and relative humidity were 23 ± 2 °C and 60 ± 5%, respectively, and soil was also aerated. All these parameters, temperature, humidity and aeration, were periodically assessed throughout the entire period of the biodegradation process which lasted 6 month. After that time film samples were carefully removed from soil and analyzed following the experimental protocol.

### 2.5. Characterization of Materials

#### 2.5.1. Particle Size Distribution and Porous Properties of Fillers

Zetasizer Nano ZS (0.6–6000 nm) (Malvern Instruments Ltd., Malvern, UK) using the non-invasive backscattering technique was applied to determine particle size and the dispersive properties of the silica–lignin samples. Size of pores were determined using an ASAP 2020 instrument (Micromeritics Instrument Co., Norcross, GA, USA).

#### 2.5.2. Differential Scanning Calorimetry

Thermal properties of materials in form of films were evaluated using DSC (DSC 1, Mettler Toledo, Greifensee, Switzerland) under argon atmosphere. For nonisothermal crystallization investigations, the samples were first heated from 40 °C to 210 °C at the rate 20 °C/min and kept at this temperature for 4 min to eliminate the previous thermal and/or mechanical history. Then the samples were quenched to 40 °C at the rate 5 °C/min. This procedure was repeated two times and the second tour was used in the calculations. Based on the determined values for the enthalpy of crystallization (*H*), the extent of crystallization (crystal conversion), α was calculated (Equation (1)).

(1) $$\alpha =\frac{\int_{0}^{t}\left(\frac{dH}{dt}\right)\times dt}{\int_{0}^{1}\left(\frac{dH}{dt}\right)\times dt}$$

From the curves of *α* = *f*(*t*), the half-time of crystallization (*t*_0.5_) was determined as time when crystal conversion was 50%. The crystallinity degree (*X_c_*) of materials was evaluated according to Equation (2).

(2) $$X_{c}=\left(\frac{\Delta H_{m}}{\Delta H_{m}{}^{\circ}\times \left(1-\frac{\%\mathrm{wt}\;\mathrm{filler}}{100}\right)}\right)\times 100$$

where: Δ*H_m_* is the melting enthalpy (from second heating scan), Δ*H_m_*° is the melting enthalpy of a 100% crystalline polymer matrix (93.0 J/g for PLA [23]) and %wt filler is the filler weight percentage. Furthermore, characteristic temperatures—melting (*T*_m_) and crystallization (*T*_c_) temperatures—were defined.

#### 2.5.3. Thermogravimetric Analysis

A Jupiter STA analyzer (Jupiter STA 449F3, Netzsch, Selb, Germany) was used to investigate the influence of filler type on thermal stability of the composites. Measurements were conducted in the atmosphere of nitrogen (flow rate 20 cm^3^/min) at a heating rate of 10 °C/min over a temperature range of 30–800 °C, with an initial sample weight of approximately 5 mg.

#### 2.5.4. Colorimetric Analysis

Colorimeter (Testan DT-145, Anticorr, Gdańsk, Poland) was used to measure differences in colors of films before and after biodegradation. Method CIE76 was applied and the differences were calculated using Equation (3).

(3) $$\Delta E_{ab}*=\sqrt{{\left(L_{2}*-L_{1}*\right)}^{2}+{\left(a_{2}*-a_{1}*\right)}^{2}+{\left(b_{2}*-b_{1}*\right)}^{2}}$$

where *L*_1_, *a*_1_, and *b*_1_ stand for the measured parameters of the standard color, whereas *L*_2_, *a*_2_, and *b*_2_ stand for the parameters of the sample. Value ∆E_ab_*~2.3 corresponds to a just noticeable difference [24].

#### 2.5.5. Tensile Properties

Tensile properties of produced composite films were defined using Zwick and Roell Allround-Line Z020 TEW testing machine (Wrocław, Poland). Samples of 10 mm width and thickness ca. 100 µm were tested with speed 5 mm/min and initial force 0.2 N in accordance to standard ISO 527-3.

#### 2.5.6. Scanning Electron Microscopy

The morphology of samples was observed using a scanning electron microscopy (EVO40, Zeiss, Jena, Germany), at acceleration voltage of 18 kV. Before testing, all the specimens were sputter-coated with gold for 5 s using a Balzers coater (PV205P, Oerlikon Balzers Coating SA, Brügg, Switzerland).

## 3. Results and Discussion

### 3.1. Characterization of Hybrid Fillers

In Table 1, particle size distributions, as well as average size of pores of hybrid fillers and its precursors are presented. The effectiveness of hybrid filler formation was already proved and discussed in our earlier paper [20]. Sizes of silica particles were in two ranges: 39–71 nm and 1440–4800 nm. In case of lignin particles no particles smaller than 100 nm were observed, lignin sample consisted of particles with 1720–5560 nm diameters. For each hybrid filler particle diameters were found to have two ranges, which were a resultant of presence of both precursors, lignin and silica. Fillers containing high amount of silica, e.g., filler 5:1, were characterized with smaller particles than fillers with high content of lignin (e.g., filler 1:1). Similar relationship can be found while considering the average size of pores. Silica had pores three times smaller than lignin (3.9 nm versus 12.3 nm). Diameter of pores of hybrid filler 20:1, containing definitely more silica than lignin, were comparable to those of silica itself. With the increasing amount of lignin in hybrid filler, the mean size of pores also increased. 

### 3.2. Differential Scanning Analysis

**Figure 1:**
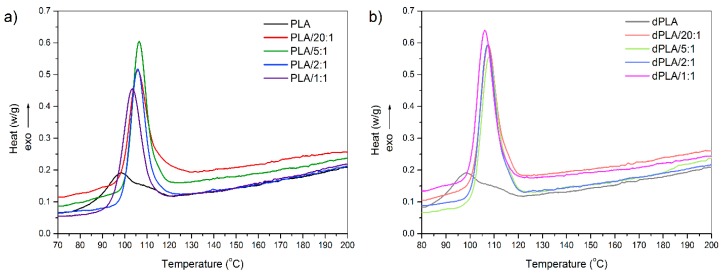
Differential scanning calorimetry (DSC) curves measured during the second cooling of samples: (**a**) before biodegradation and (**b**) after biodegradation.

It is known that introduction of filler into a polymer matrix may affect its crystallization behavior. Defining the nucleating abilities of PLA matrix in presence of hybrid fillers after biodegradation process is believed to be important in terms of defining the biodegradation mechanism of such composites. Therefore, DSC technique was applied to investigate kinetic parameters of PLA crystallization in composites. Figure 1a shows the DSC curves of the PLA and composite materials before biodegradation. Figure 1b presents analogical curves of samples subjected to simulated biodegradation. Characteristic temperatures, *X_c_* and *t*_0.5_ presented in Table 2 were calculated based on data obtained from DSC measurements.

For samples that were not subjected to simulated biodegradation the *T*_m_ was in range 174.5–177.4 °C, while for the biodegraded samples *T*_m_ was in range 175.9–177.0 °C. It is known that small changes in *T*_m_ of composites are caused by the perfection of spherulite structure of PLA. *T*_m_ values obtained in this study were very comparable and consistent with the literature [2,25]. 

The peaks at 98–108 °C are attributed to crystallization of PLA matrix. The crystallization temperature provides information about the nucleation ability of PLA in presence of hybrid fillers. In case of all, non- and biodegraded, samples addition of hybrid filler was responsible for shifting of DSC exothermic peak towards higher temperature. In comparison with unfilled PLA, *T*_c_ of composite samples before biodegradation markedly increased (by 5.5–8.5 °C). For biodegraded samples that difference in *T*_c_ was even slightly higher, and reached 8–10 °C. Obtained results are compliant with changes in *T*_c_ reported for PLA/lignin [26] and PLA/graphene composites [27]. Given the above, it can be stated that hybrid fillers act as heterogeneous nucleation agents of PLA matrix. 

The calculated *X*_c_ was in range from 47% to 60% for pristine films and from 49% to 66% for degraded samples. Among both, non- and biodegraded samples, the highest value of *X*_c_ parameter was noted for the unfilled PLA. However, samples after biodegradation were characterized with slightly higher values of *X*_c_. Similar tendency was observed by Zimmermann et al. [28]. According to literature this is probably a result of biodegradation process that in the first stage takes place in amorphous regions and thus enhances the crystallinity degree [29].

Nucleation effect and polymer chain mobility—these are two competing processes that take place during crystallization process. It is likely that introduction of hybrid filler into PLA matrix intensifies the first stage of crystallization, formation of nuclei, but at the same impairs the chain mobility. 

**Figure 2:**
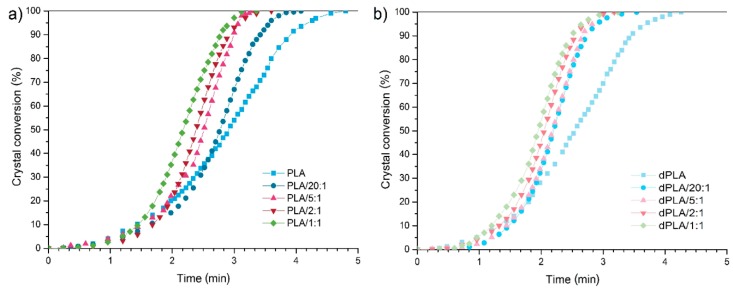
Crystal conversion curves of samples: (**a**) before biodegradation and (**b**) after biodegradation.

The analysis of crystal conversion curves (Figure 2) and crystallization half-times (Table 2) reveals that incorporation of each type of hybrid filler caused a drop of *t*_0.5_. In case of non-degraded samples, the lowest noted value of *t*_0.5_ was 2.2 min (2.9 min for pristine PLA). Silica, the main constituent of our hybrid fillers, is a well-known nucleating agent for PLA [30,31]. Generally, it is believed that as the particle size of filler decreases, its surface area increases providing more nucleating sites. However, our findings do not support the thesis that decrease of particle size (the higher was the amount of silica in hybrid filler, the smaller were particles of filler) enhances the crystallization of PLA. 

It seems rather that the composition of the hybrid filler was a major factor influencing the *t*_0.5_ parameter, especially in non-degraded composites. It was shown that crystallization half-times of PLA matrix in presence of hybrid fillers depend mainly on mobility of polymer chains. Size and surface area of fillers are crucial in terms of nucleating processes. Nonetheless, hybrid fillers with highly active surface may induce segmental limitation of polymer chains mobility, and in result, formation of spherulitic structures. It is likely that in case of PLA/hybrid filler composites the amount of small nuclei formed in matrix by silica particles was so high that it restricted the mobility of polymeric chains and consequently limited the formation of spherulites. Another factor possibly restricting the mobility of PLA chains was size of pores of the filler. Pores of 20:1 filler, containing high amounts of silica, were definitely smaller than in case of 1:1 hybrid filler (4.1 nm and 8.0 nm, respectively). Even though presence of these small pores is responsible for formation of transcrystalline layers in PLA/silica composites [20], it also hindered the movements of polymer chains. That explains why the *t*_0.5_ parameter was lower for PLA/1:1 than for PLA/20:1 films. Surprisingly, not only silica was an agent hindering the formation of PLA spherulites. As it can be seen in Table 2, crystallization half-times of all biodegraded composites were rather similar and lower than those for non-degraded films (minimal value 2.1 min for dPLA/2:1, 2.5 min for dPLA). These comparable results provide information about the course of the biodegradation process of hybrid filler. Most likely products of decomposition of lignin, constituent of hybrid filler, act as a nucleating agent for PLA matrix. It is confirmed by differences in *X*_c_ of biodegraded samples, which for composite with 1:1 hybrid filler reached 57% and for films with 20:1 hybrid filler only 49%. Here, removal of the lignin from the PLA matrix (during the biodegradation) was also responsible for the fact that PLA polymer chains regained some of their mobility.

### 3.3. Thermal Stability of Composites

**Figure 3:**
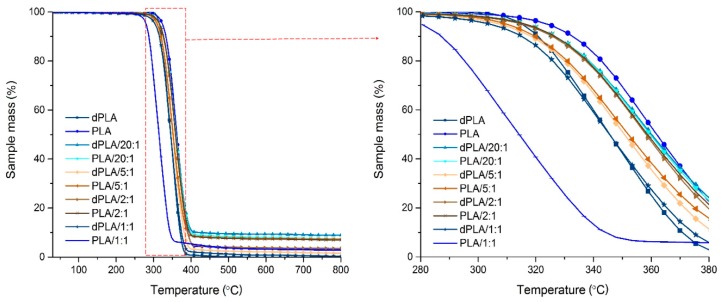
Curves for PLA-based films.

The curves of PLA-based composites with hybrid fillers are given in Figure 3.

Curves in Figure 3 indicate that the thermal degradation of PLA and PLA/hybrid filler is a rather simple process. According to Kopinke et al. [32] who performed analysis and pyrolysis-MS of PLA, at temperature 295 °C lactide is released, whereas at 350 °C also higher cyclic oligomers are removed. The curves of silica–lignin hybrid filler showed that decomposition of such material takes places in three stages—first one is associated with loss of water, during second stage (210–600 °C) intensive sample mass loss (~40%) occurs, and in the last stage fragmentation and final degradation takes place [3]. The main weight loss step for both, pristine PLA and hybrid filler, occurs in similar temperature range (280–380 °C). It is possible that because of overlapping for PLA/hybrid filler composites only one stage weight loss was observed. The tabulated values obtained from these measurements are provided in Table 3. The results indicate that biodegradation of pristine PLA causes a decrease in its thermal stability—50% wt. loss for PLA and dPLA was observed at 361 °C and 345 °C, respectively. Decrease of thermal stability (from *T*_d_ = 351.5 °C to *T*_d_ = 339.6 °C) of PLA biodegraded for 6 months was also reported by Pinto et al. [33]. 

Gordobil et al. reported that incorporation in PLA acetylated kraft lignin, as well as unmodified one, improves the thermal stability of composites [34]. However, the presence of untreated lignin in PLA composites was also shown to have a negative impact on their thermal stability [35]. 

Analysis of thermal values reveals no substantial differences in characteristic temperatures of non- and biodegraded samples containing 20:1, 5:1, and 2:1 hybrid filler. These values were also comparable with those noted for pristine PLA. However, it turned out that the difference in thermal stability of dPLA/1:1 and PLA/1:1 was particularly important. The starting PLA/1:1 sample contained relatively high amount of lignin that was probably decomposed during biodegradation. In such case silica became the main constituent of the filler and thus contributed to increase of thermal stability of composites. Given that it can be assumed that in silica–lignin hybrid fillers the presence of silica is responsible for maintaining thermal stability of composites and the lignin is a constituent. 

### 3.4. Colorimetric Analysis

**Figure 4:**
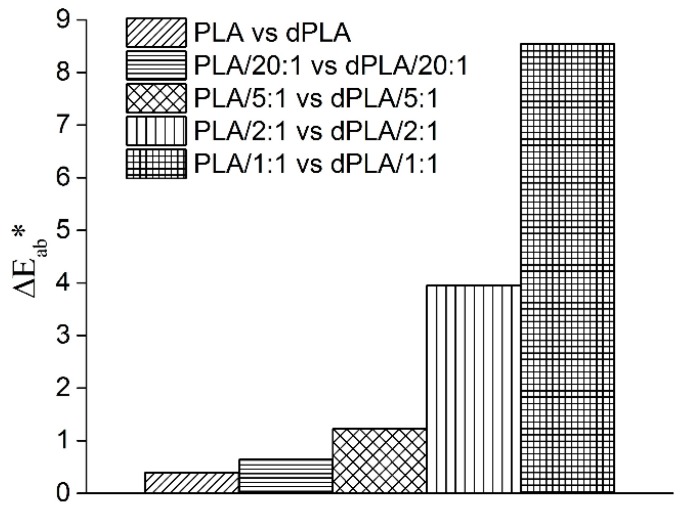
Values of ΔE_ab_* parameter for tested films.

In view of potential applications of PLA/hybrid fillers composites not only thermal and mechanical properties are important. Appearance of such films is also an essential factor. Therefore, in Figure 4 values of ΔE_ab_* parameter obtained by comparison of samples before and after biodegradation process are presented. 

Since value ∆E_ab_*~2.3 corresponds to a just noticeable difference it can be stated that only for samples of PLA/1:1 and PLA/2:1 a clearly visible change in color of films could be observed. In these samples the amount of lignin was the highest. These relationships are understandable—the lignin is known to change color not only during its isolation from biomass but also during biodegradation because of degradation of aromatic structures toward oligomeric chromophores [36,37]. This change of color, browning, is thought to be the main obstacle for high value-added use of lignin in areas such as sunscreen or dyestuff dispersants [37].

### 3.5. Mechanical Properties

Table 4 presents parameters obtained during tensile testing of primary PLA films and samples subjected to simulated biodegradation.

As it should be expected, biodegradation of composites caused a decrease of TS and YM of films. Especially dPLA/2:1 and dPLA/1:1 films were so degraded that it was impossible to test more than one sample. Incorporation of hybrid filler of any type caused an impairment of all the tested parameters. The highest values of YM, TS, and EB were obtained for neat PLA. Maximal values of TS and YM for pristine PLA were definitely higher than those for some composites. What is more, TS and YM of PLA matrix were unaffected by the biodegradation process. Similar relationship for TS was described by Karamanlioglu et al. [38] who studied influence of biotic and abiotic factors on the rate of degradation of PLA samples buried in compost and soil. TS and YM of non-degraded composite samples were similar (approximately 28–38 MPa for TS and 1.12–1.34 GPa for YM) but lower than for pristine PLA. Obtaining a good interaction between components of composites is needed for effective stress transfer between matrix and filler. Accordingly, it is crucial in terms of enhancing mechanical properties of composites [39]. In general it is believed that the increase of particle size of reinforcement causes an enhanced debonding of the filler from polymer matrix [40]. However, in this research the influence of particle size on mechanical properties of films turned out to be rather negligible. Here, the decrease in tensile properties should be rather ascribed to presence of lignin, what was also reported elsewhere [41]. Even though lignin and PLA, due to their ability to form hydrogen bonds, offer quite good compatibility in order to observe significant increase of mechanical properties fractionation or modification of lignin is often needed [42,43]. 

Values of TS and YM calculated for biodegraded samples were divergent but there is some relationship between type of filler used and tested parameters. It can be noticed that the higher was the amount of lignin in filler, the lower values of TS and YM were obtained. In order to fully understand this relationship, one has to know that two main mechanisms of biodegradation are distinguished: *(i)* bulk erosion, when water diffuses rapidly between polymer chains, causing hydrolysis and *(ii)* surface erosion during which the polymer resorbs water from its outer surface toward its center [44]. It was found that PLA composites are first being hydrolyzed in whole bulk of the material [45] and then they may undergo surface erosion [46]. It was also proved that presence of hydroxyl groups in composite fillers enables penetration of water into ester groups of PLA matrix and facilitates biodegradation process [18]. Also hydroxyl groups present in kraft lignin can contribute to its biodegradation. It is most likely that as the amount of lignin in composite increases, the water adsorption increases and thus biodegradation accelerates. That is also consistent with other research [47].

The values of the EB parameter of the evaluated samples were significantly different. PLA generally suffers from a low deformation at break thus requires the use of plasticizers [48]. However, in this case the highest value of this parameter (ca. 46%) was noted for neat PLA. Incorporation of filler in PLA matrix resulted in decrease of EB. This is a typical behavior for some composites, especially nanocomposites. Again, biodegraded composites were found to have more uniform values of EB (ca. 1–2%) than non-biodegraded samples (ca. 4–20%). EB parameter for pristine PLA was the parameter affected by the biodegradation the most. While in terms of YM and TS of both PLA samples there was almost no difference, for EB for PLA reached 46%, whereas for degraded PLA it was only 12%.

### 3.6. Morphology of Films

**Figure 5:**
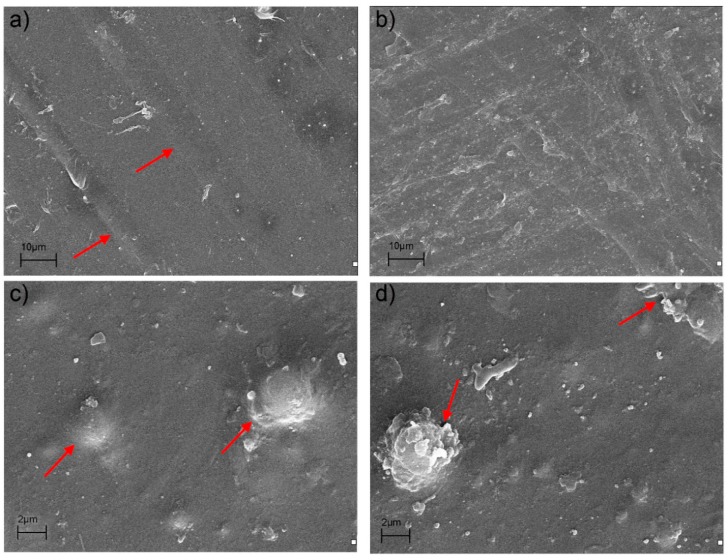
SEM images of composite films: (**a**) PLA, (**b**) dPLA, (**c**) PLA/5:1, and (**d**) dPLA/5:1.

SEM micrographs for non- and biodegraded samples (see Figure 5) were taken in order to define the morphological structure of composite films and thus provide better understanding of results of mechanical tests. 

Comparison of SEM pictures of non- and biodegraded PLA samples shows some interesting differences. On the contrary to dPLA sample (Figure 5b), the micrograph of non-degraded PLA (Figure 5a) reveals the presence of well visible parallel lines (marked with red arrows) which are the remains of orientation process occurring during extrusion of films. Orientation and polymer chain relaxation, these are two important effects that can have an impact on mechanical properties of materials. Yu et al. [49] have shown that during orientation of films under the different drawing speeds modulus and tensile strength remained almost the same, but an elongation decreased. Increased chain relaxation along with lower degree of orientation occurred during biodegradation of PLA films seem to be a reason for such significant decrease of EB parameter (from ca 46% for PLA to ca. 12% for dPLA).

In presented SEM microphotographs both, lignin and silica particles can be observed. Figure 5c,d reveal the presence of some aggregates of silica (seen as light points) with particles sizes below 1 µm as well as some bigger silica aggregates (ca. 2 µm) covered with polymer matrix. On the other hand, lignins are known to have spherical shape which is a result of their three-dimensional structure [50]. An example of such big, well-defined, spherical lignin particle, fully covered with PLA matrix, can be seen in Figure 5c. That sample was also characterized with good interphase adhesion between polymer matrix and particles of the filler. On the contrary, as shown in Figure 5d, the surface of the degraded sample was less uniform when compared to non-degraded sample. The PLA matrix itself remained intact, but the lignin particles were already partially decomposed, showing some delamination. What is crucial, in the biodegraded composite film the lignin particles and products of their degradation were almost entirely uncovered with polymer. Also some cracks at the matrix/filler interphase were present. These cracks were responsible for lowering the mechanical properties of composites subjected to biodegradation. According to literature, hydroxyl groups present in lignin are partially responsible for its biodegradation [7]. Moreover, weight loss studies have shown that samples containing high amount of lignin underwent the highest weight loss—7.7% and 6.5% for composites with 1:1 and 2:1 hybrid filler, respectively. Weight loss for other samples was as follows: 0.9% for unfilled PLA, 2.8% for PLA with 20:1 hybrid, and 4.3% for PLA filled with 5:1 hybrid. Therefore, it is believed that in PLA/hybrid filler films lignin was an ingredient that underwent biodegradation as first, causing a discontinuity of PLA matrix. 

## 4. Conclusions

The subject of this study was to prepare PLA/hybrid fillers composites and determine changes taking place during biodegradation of PLA. Measurements were taken so to define thermal stability and crystallization behavior, while morphology of samples was investigated by SEM technique. Changes in color and mechanical properties of composites were examined as well. All of these methods were used to investigate both, non- and biodegraded samples. To the best knowledge of the authors this is the first publication regarding biodegradation of PLA composites with silica–lignin hybrids as fillers. 

It was found that thermal studies, including phase transitions observations, can provide information about the course of biodegradation process of PLA/hybrid filler composites. The results show that during the biodegradation of hybrid fillers lignin is a component that undergoes degradation as first. Products of its decomposition turned out to act as heterogeneous nucleation agent for PLA. However, the major agent determining thermal stability of composites was the presence of silica. Susceptibility to biodegradation and thermo-mechanical properties of prepared composites are strongly related to composition of hybrid filler. Composites with hybrid filler containing high amount of lignin were the most active in terms of nucleating abilities. However, such composites were characterized with the lowest thermal stability. These findings suggest that composition of hybrid fillers should be further optimized. Obtained results indicate that composite with 2:1 hybrid filler was the most thermally stable (comparable to unfilled PLA) and underwent biodegradation process the most effectively.

## Figures and Tables

**Table 1:** Particle size distribution and mean size of pores for hybrid fillers and their precursors.

Sample	Particle Size Distribution Range (nm)	Mean Size of Pores (nm)
Silica	39–71; 1440–4800	3.9
Lignin	1720–5560	12.3
Filler 20:1	39–79; 1720–4800	4.1
Filler 5:1	68–122; 1990–4800	4.8
Filler 2:1	79–220; 1720–4800	5.5
Filler 1:1	91–220; 1990–5560	8.0

**Table 2:** Tabulated values of melting temperatures (*T*_m_), crystallization temperatures (*T*_c_), crystallinity degree (*X_c_*), and half-time of crystallization (*t*_0.5_) of samples before and after biodegradation.

Sample	*T*_m_ (°C)	*T*_c_ (°C)	*X_c_* (%)	*t*_0.5_ (min)
PLA	177.0	98.0	60	2.9
PLA/20:1	177.4	105.9	47	2.8
PLA/5:1	176.9	106.6	48	2.5
PLA/2:1	175.9	105.8	51	2.4
PLA/1:1	174.5	103.5	52	2.2
dPLA	176.8	98.1	66	2.5
dPLA/20:1	177.0	107.6	49	2.2
dPLA/5:1	177.0	107.9	51	2.2
dPLA/2:1	176.9	107.2	55	2.1
dPLA/1:1	175.9	106.1	57	2.0

**Table 3:** Tabulated thermal analyses values of tested films.

Sample	5% wt. Loss	15% wt. Loss	50% wt. Loss
	Temperature (°C)		
PLA	326	337	361
PLA/20:1	314	331	359
PLA/5:1	303	326	350
PLA/2:1	314	332	358
PLA/1:1	281	291	315
dPLA	314	326	345
dPLA/20:1	314	331	360
dPLA/5:1	303	325	350
dPLA/2:1	314	332	356
dPLA/1:1	280	321	345

**Table 4:** Young’s modulus (YM), tensile strength (TS), and elongation at break (EB) parameters calculated for PLA films before and after biodegradation.

Sample	YM (GPa)	TS (MPa)	EB (%)
PLA	2.08 ± 0.12	52.28 ± 5.59	46.33 ± 17.17
PLA/20:1	1.15 ± 0.18	27.94 ± 1.84	10.23 ± 1.20
PLA/5:1	1.33 ± 0.13	30.42 ± 2.56	20.37 ± 7.20
PLA/2:1	1.34 ± 0.21	38.17 ± 1.08	5.26 ± 1.38
PLA/1:1	1.12 ± 0.18	30.07 ± 3.14	3.67 ± 0.79
dPLA	2.11 ± 0.55	52.93 ± 2.53	12.30 ± 3.75
dPLA/20:1	0.67 ± 0.11	21.30 ± 0.25	1.75 ± 0.04
dPLA/5:1	0.37 ± 0.15	13.39 ± 6.12	2.01 ± 0.68
dPLA/2:1	0.29 *	7.58 *	1.67 *
dPLA/1:1	0.14 *	3.17 *	1.41 *

For samples marked with ‘*’ standard deviation was not calculated.
